# Determining Split Renal Function in Children With Ureteropelvic Junction Stenosis: Technetium-99m Mercaptoacetyltriglycine (Tc-99m MAG-3) or Technetium-99m Dimercaptosuccinic Acid (Tc-99m DMSA)?

**DOI:** 10.7759/cureus.65075

**Published:** 2024-07-22

**Authors:** Hale Özdemir, İlknur Girişgen, Olga Yaylalı, Tülay Becerir, Özkan Herek, Hande Şenol, Selçuk Yüksel

**Affiliations:** 1 Department of Pediatrics, Bingöl Genç State Hospital, Bingöl, TUR; 2 Department of Pediatric Nephrology, Pamukkale University Faculty of Medicine, Denizli, TUR; 3 Department of Nuclear Medicine, Pamukkale University Faculty of Medicine, Denizli, TUR; 4 Department of Pediatric Surgery, Pamukkale University Faculty of Medicine, Denizli, TUR; 5 Department of Biostatistics, Pamukkale University Faculty of Medicine, Denizli, TUR; 6 Department of Pediatric Rheumatology and Nephrology, Çanakkale Onsekiz Mart University, Faculty of Medicine, Çanakkale, TUR

**Keywords:** tc-99m dmsa, tc-99m mag-3 scintigraphy, children, hydronephrosis, ureteropelvic junction stenosis

## Abstract

Background

Ureteropelvic junction stenosis (UPJS) is the most common cause of clinically significant antenatal hydronephrosis. We compared separate renal function results obtained using technetium-99m-mercaptoacetyltriglycine (Tc-99m MAG-3) and technetium-99m-dimercaptosuccinic acid (Tc-99m DMSA) in pediatric patients with UPJS to evaluate the adequacy of Tc-99m MAG-3 scintigraphy and the necessity of additional Tc-99m DMSA scintigraphy during follow-up.

Methodology

Patients diagnosed with hydronephrosis in the Pediatric Nephrology Department of Pamukkale University Faculty of Medicine over a period of 10 years (2012-2022) were evaluated retrospectively. Patients who had been diagnosed with UPJS and underwent both Tc-99m MAG-3 and Tc-99m DMSA scintigraphy during follow-up were included in the study. Technetium-99m-labeled MAG-3 and DMSA scans were re-evaluated for all patients by the Department of Nuclear Medicine.

Results

The study included 52 children with unilateral UPJS (12 girls and 40 boys) with a mean age of 6.34 ± 4.81 years (range: 2.97-9.79 years). Thirty-six patients (69.2%) were diagnosed antenatally. Differential renal function in Tc-99m DMSA was 46.94 ± 10.64 and in Tc-99m MAG-3 was 43.08 ± 11.18; the functions were lower in Tc-99m MAG-3, but the values were within normal limits for both groups (p=0.0001, z=-3.893). When differential renal functions were compared between Tc-99m DMSA and Tc-99m MAG-3 results, a statistically significant positive and strong correlation was found in the kidney with ureteropelvic junction obstruction (UPJO) (p=0.0001, r=0.752).

When classifying the Tc-99m MAG-3 and Tc-99m DMSA results in the kidney with UPJO (supranormal, normal, low function) for the determination of differential renal functions, there was a consistency of 76%, and it was correlated (p=0.0001, k=0.456). While two patients had supranormal function and 13 patients had low function in Tc-99m MAG-3, five patients had supranormal function, and eight patients had low function in Tc-99m DMSA.

Conclusions

Some studies in the literature have reported that Tc-99m MAG-3 causes supranormal function measurements in patients with UPJS; our results showed that Tc-99m DMSA resulted in a higher rate of supranormal values for affected kidneys. We believe that Tc-99m DMSA should not be performed in addition to Tc-99m MAG-3 scintigraphy in the follow-up of every patient with UPJS but can be utilized in select cases, such as patients with surgical indications and those suspected before surgery.

## Introduction

Ureteropelvic junction stenosis (UPJS) is the most common cause of clinically significant antenatal hydronephrosis [1,2]. This condition can lead to permanent kidney damage. The management of UPJS may be surgical or conservative follow-up, depending on the condition of the kidney [3]. Although certain criteria are used to decide whether to follow up conservatively or perform surgery (timing and indications), there is no clear guideline. However, important indications for surgical treatment include the degree of hydronephrosis on ultrasonography (USG), findings of scarring, an obstructive pattern, or split renal function (SRF) measurement below 40% on scintigraphy, or more than a 10% reduction in SRF in scintigraphy follow-up [4].

The first-line imaging method in UPJS for diagnosis, follow-up, and treatment decision-making is USG [5-7]. In addition, technetium-99m-mercaptoacetyltriglycine (Tc-99m MAG-3) and technetium-99m-dimercaptosuccinic acid (Tc-99m DMSA) scintigraphy, two nuclear medicine imaging methods, are also used for the same purposes [1,6-8]. Unlike radiological imaging methods, scintigraphic methods enable the calculation of SRF [9]. SRF indicates the distribution of total renal function between the two kidneys [10]. Although renal function is reduced in UPJS, measured SRF values can sometimes be higher than normal, which is termed supranormal function (>55%) [7,10-12]. Supranormal function measurements result in SRF that is clinically too low to be evaluated as good, leading to delayed surgical treatment decisions. Furthermore, if SRF decreases to its true low value after surgery in a kidney with supranormal function, this may be interpreted as a postoperative loss of function and can lead to questioning of the surgical treatment.

Some studies have suggested that Tc-99m MAG-3 scintigraphy overestimates SRF in UPJS, while Tc-99m DMSA scintigraphy provides more accurate SRF measurements [5,7,13]. In contrast, other studies have indicated that Tc-99m MAG-3 provides accurate SRF measurements comparable to Tc-99m DMSA results and thus can prevent unnecessary radiation exposure with extra Tc-99m DMSA imaging in children [11,14]. In the present study, we compared SRF results obtained from Tc-99m MAG-3 and Tc-99m DMSA in pediatric patients with UPJS to evaluate the adequacy of Tc-99m MAG-3 scintigraphy and the necessity of additional Tc-99m DMSA scintigraphy during follow-up.

## Materials and methods

Intervention and data collection

Patients diagnosed with hydronephrosis in the Pediatric Nephrology Department of Pamukkale University Faculty of Medicine over a period of 10 years (2012-2022) were evaluated retrospectively. Patients who had been diagnosed with UPJS and underwent both Tc-99m MAG-3 and Tc-99m DMSA scintigraphy during follow-up were included in the study. Children with bilateral hydronephrosis, vesicoureteral reflux (VUR), ureterovesical junction (UVJ) stenosis, horseshoe kidney, or multicystic dysplastic kidney were excluded. A total of 52 patients meeting these criteria were included in the study.

Demographic and clinical data such as age at diagnosis, sex, follow-up period, laboratory findings, and history of urinary tract infection, stones, nephrostomy, and surgery were collected from the records of the Pediatric Nephrology Department. For patients with a surgical history, preoperative imaging was evaluated. Kidneys with UPJS were compared to normal kidneys on the opposite side according to USG and scintigraphy findings. USG results were analyzed for kidney size, size difference between the kidneys, anterior-posterior (AP) diameter, parenchymal thinning, and increased parenchymal echogenicity. A difference of more than 10 mm between the size of the normal kidney and the kidney with UPJS was defined as a significant size difference. We also evaluated whether there was an increase in kidney size according to patient age and height [15]. Hydronephrosis was evaluated as mild at AP diameters <15 mm, moderate at 15-30 mm, and severe at diameters >30 mm [6,16,17]. Parenchymal thickness <7 mm was defined as renal parenchymal thinning [18,19]. Hydronephrosis was also graded using the Society for Fetal Urology (SFU), Urinary Tract Dilatation classification (UTD), and Onen grading systems based on USG findings.

Technetium-99m-labeled MAG-3 and DMSA scans were performed and re-evaluated on all patients by the Department of Nuclear Medicine at Pamukkale University Faculty of Medicine. Static images were taken 2-3 hours after the IV administration of Tc-99m DMSA to pediatric patients by adjusting the dose to be between 15 MBq and 110 MBq according to the patient's body surface. For Tc-99m MAG-3, simultaneous dynamic imaging was performed by administering IV doses between 15 MBq and 70 MBq according to the patient's body weight. The activity to be injected is based on the European Association of Nuclear Medicine (EANM) pediatric dosage card [20,21]. Before static and dynamic imaging, all patients were hydrated orally to reduce pelvic retention by increasing diuresis, and to prevent poor excretion and thus erroneous interpretations. For this purpose, we found oral hydration to be sufficient, and there was no need for IV fluid support. To calculate SRF, regions of interest (ROI) curves were drawn around the renal parenchyma on AP images.

SRF values obtained from Tc-99m MAG-3 and Tc-99m DMSA imaging were compared between UPJS-affected and normal kidneys. Additionally, scar formation, reduced renal perfusion, and elimination half-life were evaluated. SRF on Tc-99m MAG-3 and Tc-99m DMSA was classified as supranormal (>55%), normal (40%-55%), or low (<40%) [6,22-24]. A ≥10% difference in SRF values was defined as a significant difference in function between the kidneys [25]. Tc-99m MAG-3 and Tc-99m DMSA results were compared to assess their agreement in measuring supranormal, normal, and low function. USG findings (hydronephrosis staging, kidney size, size difference between the kidneys, AP diameter, parenchymal thinning, and parenchymal echogenicity) were also compared with the results of scintigraphic imaging.

Statistical analysis

Data were analyzed using SPSS version 25.0 software. Continuous variables were expressed as means, SDs, medians, interquartile ranges (IQR; 25th-75th percentiles), and ranges (minimum-maximum values). Categorical variables were expressed as frequencies and percentages. The Kolmogorov-Smirnov and Shapiro-Wilk tests were used to determine whether the data were normally distributed. Independent samples t-tests were used for comparisons among groups. Chi-square tests were used to compare categorical variables. Spearman correlation analysis was used to examine the relationships between continuous variables. For pairwise comparisons, the Wilcoxon signed-rank test was used. Additionally, to demonstrate the compatibility of DMSA and MAG3 examinations, we used frequencies and percentages of individuals who were compatible, along with the kappa coefficient. In all analyses, a p-value of <0.05 was considered statistically significant.

## Results

The study included 52 children with unilateral UPJS (12 girls and 40 boys) with a mean age of 6.34 ± 4.81 years (range: 2.97-9.79 years). The follow-up period was 2.46 years (1.27-4.3 years). Thirty-six patients (69.2%) were diagnosed antenatally. Stones were detected in seven patients (13.5%), and 33 (63.5%) had a history of urinary tract infection. Twenty-two patients (42.3%) underwent surgery, while the other 30 patients had conservative follow-up. A nephrostomy catheter was inserted in five patients (9.6%). Microalbuminuria was present in 14 (28.6%) patients.

USG showed that UPJS-affected kidneys had significantly increased kidney size and AP diameter, and significantly lower parenchymal thickness than normal kidneys (Table 1).

**Table 1:** Ultrasonography findings of kidneys with ureteropelvic junction stenosis and normal kidneys. *p<0.05 statistically significant. AP diameter: Anterior-posterior diameter.

		Hydronephrosis	Normal kidney	
Kidney size	A.O ± S.S	80.08 ± 23.05	70.29 ± 19.75	p = 0.0001* t = 5.223
	Med (IQR)	75 (65-89.75)	65 (55 - 81)	
Parenchymal thickness	A.O ± S.S	6.81 ± 3.71	11.35 ± 2.74	p = 0.0001* t = -8.255
	Med (IQR)	5.7 (4-9.75)	11.75 (9.75-13.13)	
AP diameter	A.O ± S.S	20.86 ± 10.65	<7mm	
	Med (IQR)	20 (14-26)	<7mm	

Thirty-nine patients (75.0%) had UPJS on the left side. Of the kidneys with UPJS, kidney size was assessed as large for age and height in 25 patients (48.1%). There was a marked difference in size (>10 mm) between the affected kidney and the normal kidney in 28 patients (53.8%). Parenchymal thinning (<7 mm) in the affected kidney was observed in 35 patients (67.3%), and increased parenchymal echogenicity was observed in 13 patients (25.0%). The increase in AP diameter in the kidney with UPJS was classified as mild in 13 patients (25.0%), moderate in 32 patients (61.5%), and severe in 7 patients (13.5%). The patients' USG findings and the results of hydronephrosis staging according to the SFU, Onen, and UTD classification systems are shown in Table 2.

**Table 2:** SFU, Onen, and UTD classification. SFU: Society for Fetal Urology; UTD: Urinary Tract Dilatation classification.

SFU		Onen		UTD	
Grade	Number of patients	Grade	Number of patients	Grade	Number of patients
1-2a	1 (1.9%)	1	2 (3.8%)	1	2 (3.8%)
2b	9 (17.3%)	2	15 (28.8%)	2	15 (28.8%)
3	7 (15.5%)	3	29 (55.8%)	3	35 (67.3%)
4	35 (67.3%)	4	6 (11.5%)		

**Figure 1:**
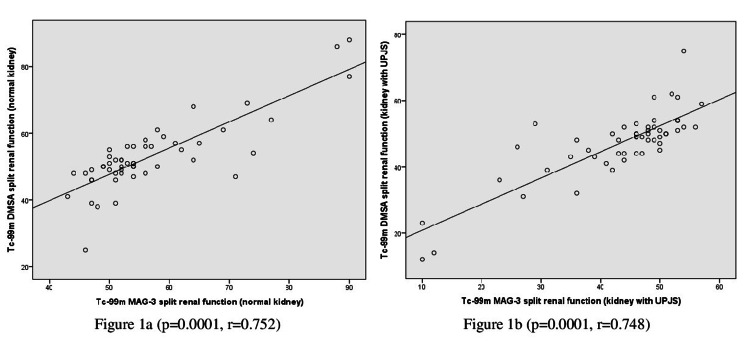
Correlation analysis of split renal functions in Tc-99m MAG-3 and Tc-99m DMSA results in kidneys with UPJS (1b) and normal kidneys (1a). Tc-99m MAG-3: Technetium-99m-mercaptoacetyltriglycine; Tc-99m DMSA: Technetium-99m-dimercaptosuccinic acid; UPJS: Ureteropelvic junction stenosis.

Differential renal function in Tc-99m DMSA was 46.94 ± 10.64 (median = 49 (44-52)) and in Tc-99m MAG-3 was 43.08 ± 11.18 (median = 46 (39.5-50)); the functions were lower in Tc-99m MAG-3, but the values were within normal limits in both groups (p=0.0001*, z=-3.893). In the comparison of SRF according to Tc-99m MAG-3 and Tc-99m DMSA, both methods indicated supranormal function in one patient (1.9%), normal function in 32 patients (61.5%), and low function in seven patients (13.5%). In the other 12 patients, SRF classifications differed between the two methods. The correlation rate was 76% (Table 3 and Figure 1). When the correlation between SRF evaluated with Tc-99m MAG-3 and Tc-99m DMSA in UPJS-affected kidneys was examined, the kappa coefficient of agreement was significant, and the results were statistically congruent (p=0.0001, κ=0.456) (Table 3). SRF values obtained with Tc-99m DMSA and Tc-99m MAG-3 showed a statistically significant positive correlation between the kidneys with UPJS and normal kidneys (Figure 1) (p=0.0001, r=0.752).

**Table 3:** Comparison of kidney function in UPJS using Tc-99m MAG3 and Tc-99m DMSA. *p<0.05 statistically significant k = Chi-square test. Tc-99m MAG-3: Technetium-99m-mercaptoacetyltriglycine; Tc-99m DMSA: Technetium-99m-dimercaptosuccinic acid; UPJS: Ureteropelvic junction stenosis.

		Tc-99m MAG3				
		Supranormal	Normal	Low	Total	
Tc-99m DMSA	Supranormal	1 (1.9%)	4 (7.7%)	0 (0.0%)	5 (9.6%)	P=0.0001* (k=0.456)
	Normal	1 (1.9%)	32 (61.5%)	6 (11.5%)	39 (75.0%)	
	Low	0 (0.0%)	1 (1.9%)	7 (13.5%)	8 (15.4%)	
	Total	2 (3.8%)	37 (71.2%)	13 (25.0%)	52 (100.0%)	

Increased kidney size on USG showed no significant correlation with SRF measured by Tc-99m MAG-3 but was significantly correlated with SRF measured by Tc-99m DMSA (p=0.018) (Table 4). All patients with supranormal function on Tc-99m DMSA had increased kidney size on USG.

**Table 4:** Comparison of size increase in kidneys with UPJS on ultrasonography and split renal function measured by Tc-99m MAG-3 and Tc-99m DMSA. *p<0.05 statistically significant; k=chi-square test. Tc-99m MAG-3: Technetium-99m-mercaptoacetyltriglycine; Tc-99m DMSA: Technetium-99m-dimercaptosuccinic acid; UPJS: Ureteropelvic junction stenosis.

		Tc-99m MAG-3 split renal function				
		Supranormal	Normal	Low	Total	
Ultrasonography kidney size increase	Yes	1 (50.0%)	20 (54.1%)	4 (30.8%)	25 (48.1%)	p=0.343 k=2.14
	No	1 (50.0%)	17 (45.9%)	9 (69.2%)	27 (51.9%)	
	Total	2 (100.0%)	37 (100.0%)	13 (100.0%)	52 (100.0%)	
		Tc-99m DMSA split renal function				
		Supranormal	Normal	Low	Total	
Ultrasonography kidney size increase	Yes	5 (100.0%)	17 (43.6%)	3 (37.5%)	25 (48.1%)	p=0.018* k=8.003
	No	0 (0.0%)	22 (56.4%)	5 (67.5%)	27 (51.9%)	
	Total	5 (100.0%)	39 (100.0%)	8 (100.0%)	52 (100.0%)	

In UPJS-affected kidneys, there was no statistically significant association between hydronephrosis grade on USG and SRF level measured by Tc-99m MAG-3 or Tc-99m DMSA. There was also no statistically significant relationship between parenchymal thinning on USG and SRF measured by Tc-99m DMSA or Tc-99m MAG-3.

Furthermore, SRF measured by Tc-99m DMSA and Tc-99m MAG-3 was not associated with SFU, Onen, or UTD classifications (Table 5). When compared with SRF measured by Tc-99m MAG-3 and Tc-99m DMSA, one of the two patients with supranormal function according to Tc-99m MAG-3 was SFU grade 2b, and the other was SFU stage 3. These two patients with supranormal function measurement were assessed as grade 2 according to the Onen system and grade 2 according to the UTD classification. Of the 5 patients with supranormal function according to Tc-99m DMSA, 4 patients were SFU grade 4 and 1 patient was SFU grade 3. When assessed using the Onen staging system, 1 of these patients was grade 4, 3 patients were grade 3, and 1 patient was consistent with grade 2. When evaluated using the UTD classification, 4 of these patients were assessed as UTD stage 3 and 1 patient as UTD stage 2.

**Table 5:** Evaluation of Tc-99m MAG3 and Tc-99m DMSA split renal functions according to staging systems calculated by ultrasonography. k = Chi-square test. SFU: Society for Fetal Urology; UTD: Urinary Tract Dilatation classification; Tc-99m MAG-3: Technetium-99m-mercaptoacetyltriglycine; Tc-99m DMSA: Technetium-99m-dimercaptosuccinic acid.

Tc-99m MAG-3 split renal function						Tc-99m DMSA split renal function				
	Supranormal	Normal	Low	Total		Supranormal	Normal	Low	Total	
SFU					p=0.196 k=8.62					p=0.653 k=4.173
1-2a	0 (0.0%)	0 (0.0%)	1 (7.7%)	1 (1.9%)		0 (0.0%)	1 (2.6%)	0 (0.0%)	1 (1.9%)	
2b	1 (50.0%)	5 (13.5%)	3 (23.1%)	9 (17.3%)		0 (0.0%)	7 (17.9%)	2 (25.0%)	9 (17.3%)	
3	1 (50.0%)	5 (13.5%)	1 (7.7%)	7 (13.5%)		1 (20.0%)	4 (10.3%)	2 (25.0%)	7 (13.5%)	
4	0 (0.0%)	27 (73.0%)	8 (61.5%)	35 (67.5%)		4 (80.0%)	27 (69.2%)	4 (50.0%)	35 (67.3%)	
Total	2 (100.0%)	37 (100.0%)	13 (100.0%)	52 (100.0%)		5 (100.0%)	39 (100.0%)	8 (100.0%)	52 (100.0%)	
Onen					p=0.192 k=8.679					p=0.404 k=6.177
1	0 (0.0%)	1 (2.7%)	1 (7.7%)	2 (3.8%)		0 (0.0%)	2 (5.1%)	0 (0.0%)	2 (3.8%)	
2	2 (100.0%)	9 (24.3%)	4 (30.8%)	15 (28.8%)		1 (20.0%)	10 (25.6%)	4 (50.0%)	15 (28.8%)	
3	0 (0.0%)	24 (64.9%)	5 (38.5%)	29 (55.8%)		3 (60.0%)	24 (61.5%)	2 (25.0%)	29 (55.8%)	
4	0 (0.0%)	3 (8.1%)	3 (23.1%)	6 (11.5%)		1 (20.0%)	3 (7.7%)	2 (25.0%)	6 (1.5%)	
Total	2 (100.0%)	37 (100.0%)	13 (100.0%)	52 (100.0%)		5 (100.0%)	39 (100.0%)	8 (100.0%)	52 (100.0%)	
UTD					p=0.197 k=6.031					p=0.560 k=2.988
1	0 (0.0%)	1 (2.7%)	1 (7.7%)	2 (3.8%)		0 (0.0%)	2 (5.1%)	0 (0.0%)	2 (3.8%)	
2	2 (100.0%)	9(24.3%)	4 (30.8%)	15 (28.8%)		1 (20.0%)	10 (25.6%)	4 (50.0%)	15 (28.8%)	
3	0 (0.0%)	27(73.0%)	8 (61.5%)	35 (67.3%)		4 (80.0%)	27 (69.2%)	4 (50.0%)	35 (67.3%)	
Total	2 (100.0%)	37(100.0%)	13 (100.0%)	52 (100.0%)		5 (100.0%)	39 (100.0%)	8 (100.0%)	52 (100.0%)	

In comparison of parenchymal thinning with USG and Tc-99m MAG3; showed that 26 (83.9%) of 35 patients with parenchymal thinning on USG also had parenchymal thinning according to Tc-99m MAG-3 (Table 6).

**Table 6:** Comparison of parenchymal thinning in ultrasonography and Tc-99m MAG-3 results. *p<0.05 statistically significant k = Chi-square test. Tc-99m MAG-3: Technetium-99m-mercaptoacetyltriglycine.

		Tc-99m MAG-3 parenchymal thinning			
		Yes	No	Total	p=0.002* k=9.57
Ultrasonography parenchymal thinning	Yes	26 (83.9%)	9 (42.9%)	35 (67.3%)	
	No	5 (16.1%)	12 (57.1%)	17 (32.7%)	
	Total	31 (100.0%)	21 (100.0%)	52 (100.0%)	

## Discussion

Nuclear medicine imaging methods are used in addition to USG in the diagnosis, follow-up, and treatment of UPJS [5]. However, the supranormal measurement of SRF in kidneys with UPJS can be misleading when making treatment decisions. This study aimed to compare the Tc-99m MAG-3 and Tc-99m DMSA scintigraphy methods in terms of determining SRF, which is important in surgical decision-making, and to evaluate whether follow-up with Tc-99m MAG-3 scintigraphy is sufficient and whether Tc-99m DMSA scintigraphy is necessary in the follow-up of pediatric patients with UPJS. In our study, Tc-99m MAG-3 and Tc-99m DMSA results for SRF were found to be correlated, and there was statistically significant agreement between the results.

According to the literature, UPJS is 2-3 times more common in boys than in girls [7,16,17,26]. In our study, the male-to-female ratio was 3.33, and the mean age was 6.34 ± 4.81 years (range: 2.97-9.79 years). UPJS usually presents unilaterally, and 65% of cases are on the left side [7,16]. Consistent with the literature, UPJS was more common in the left kidney in our study (75%). With the widespread use of routine USG examination during pregnancy, UPJS is among the anomalies frequently detected antenatally [18]. In our study, 36 of the patients (69.2%) were diagnosed with UPJS antenatally. Patients who were not diagnosed in the antenatal period may present for reasons such as an abdominal mass, flank pain, urinary tract infection, and kidney stones, and may be diagnosed incidentally in USG examinations [6,18,27]. Thirty-three patients in our study (63.5%) had a history of urinary tract infection. Flank pain was present in 5 patients (9.6%), and stones were detected in seven patients (13.5%). Twenty-two of the 52 patients in our study underwent pyeloplasty. Their surgical indications included SRF <40% (6 patients), more than a 10% decrease in SRF at follow-up (6 patients), increased AP diameter and parenchymal thinning (5 patients), complete obstruction on Tc-99m MAG-3 (4 patients), and obstruction and stones (1 patient).

Low SRF is one of the most important factors in treatment decisions for UPJS. Specifically, in older children (age > 2 years) with poor function (split function < 10%), the split function plays an important role in the assessment of management (pyeloplasty vs. nephrectomy). Here, the use of DMSA vs. MAG-3 would play a crucial role. Some authors have suggested that Tc-99m MAG-3 overestimates SRF because of hydronephrosis and that Tc-99m DMSA is the gold standard for accurate SRF measurement [5,7,13]. Conversely, others have reported that Tc-99m MAG-3 is comparable to Tc-99m DMSA in SRF measurement accuracy and prevents unnecessary radiation exposure and loss of time [11,14,28,29]. Akbal C et al. [25] examined the Tc-99m MAG-3 and Tc-99m DMSA results of 81 patients with hydronephrosis (posterior urethral valve, UPJS, UV stenosis) and determined that SRF values were similar with both methods. They concluded that Tc-99m MAG-3 imaging alone would be sufficient in follow-up, with Tc-99m DMSA performed in suspected cases and patients with cortical defects. Similarly, another study evaluating 56 hydronephrosis patients including all urological abnormalities (VUR, UPJS, chronic kidney disease, solitary kidney) showed that SRF values were correlated in Tc-99m MAG-3 and Tc-99m DMSA, and Tc-99m MAG-3 SRF values were higher only in 6 patients younger than 2 months of age, which was attributed to nephron immaturity [14]. The authors stated that because Tc-99m DMSA imaging is not necessary in all patients, causes more radiation exposure, and time loss for the patient, and provides comparable SRF results, Tc-99m MAG-3 can be used instead of Tc-99m DMSA [14]. Aktaş GE et al. [4] examined SRF using Tc-99m MAG-3 and Tc-99m DMSA in 19 patients with unilateral hydronephrosis and also found SRF values to be correlated in the two methods. They noted that Tc-99m MAG-3 had advantages over Tc-99m DMSA such as rapid excretion and lower radiation exposure. In our study, there was a statistically significant and strong positive correlation between Tc-99m MAG-3 and Tc-99m DMSA results for SRF in both UPJS and healthy kidneys (p=0.0001). For kidneys with UPJS, there was also 76% agreement (40 patients) in the SRF classifications (supranormal, normal, low) made by Tc-99m MAG-3 and Tc-99m DMSA (p=0.0001, κ=0.456).

Although UPJS causes impaired renal function, SRF can sometimes be measured as supranormal (>55%) in kidneys with significant stenosis [7,10,11,30]. Supranormal function has been reported in 9% to 28% of patients diagnosed with UPJS in the literature [11,31-33]. Some authors interpret these supranormal function measurements as artifactual, while others argue that the phenomenon is associated with factors such as increased volume and filtration capacity of a single nephron, reduced function in the contralateral kidney, renal immaturity, and different isotope distributions. They claimed that USG parameters such as increased AP diameter, kidney size, and the size difference between the two kidneys could predict supranormal function [7,10,11,30,34-36]. Additionally, studies suggest that SRF assessed by Tc-99m MAG-3 shows supranormal function, thus indicating that Tc-99m DMSA is more accurate in measuring SRF [5,13]. Ritchie G et al. [13] observed a difference between Tc-99m MAG-3 and Tc-99m DMSA SRF measurements and stated that Tc-99m DMSA was a better indicator of SRF measurement. However, considering the higher rate of radiation with Tc-99m DMSA and the clinically acceptable difference between the two methods in determining SRF, they concluded that Tc-99m DMSA was not required in addition to Tc-99m MAG-3, especially in patients with normal SRF and no scarring. In our study, two patients had supranormal function and 13 patients had low function on Tc-99m MAG-3, while five patients had supranormal function and eight patients had low function on Tc-99m DMSA. The frequency of supranormal function was higher with Tc-99m DMSA compared to Tc-99m MAG-3, whereas Tc-99m MAG-3 detected more low-functioning kidneys than Tc-99m DMSA. Additionally, differential renal functions were found to be lower in Tc-99m MAG-3 than in Tc-99m DMSA.

Previous studies have shown that the AP diameter and the ratio of renal pelvis volume to kidney volume are associated with supranormal function measurements [11,30]. Martín-Solé O et al. [11] found that an AP diameter of >30 mm was a strong predictive indicator of supranormal function, although in our study, AP diameter was not associated with supranormal function assessed by either Tc-99m MAG-3 or Tc-99m DMSA. However, this may have been related to our small number of supranormal cases. Conversely, in a study of adult patients, supranormal function was associated with increased renal pelvis volume, increased AP diameter, and high-grade hydronephrosis according to SFU grading [30]. In our study, there was no statistical association between the degree of renal AP diameter increase (mild, moderate, severe) and SRF level (supranormal, normal, low) according to Tc-99m MAG-3 or Tc-99m DMSA (p=0.454 and p=0.783, respectively). Additionally, there was no significant relationship between increased kidney size and SRF in Tc-99m MAG-3 according to age and height on USG. Renal size was significantly increased for age and height on USG in all kidneys measured as having supranormal function by Tc-99m DMSA. Moon DH et al. [37] evaluated hydronephrosis size on USG compared to SRF in Tc-99m MAG-3 and found that higher-grade (SFU grade 4) hydronephrosis was associated with supranormal function. A study of adult patients also showed that supranormal function was associated with increased renal pelvis volume, increased AP diameter, and higher SFU hydronephrosis grade [30]. In our study, no association was found between Onen, SFU, and UTD grade on USG and SRF values obtained by Tc-99m MAG-3 or Tc-99m DMSA. Pippi Salle JL et al. [35] stated that supranormal function is not an artifact but a physical state that occurs due to radioisotope distribution related to severe hydronephrosis and parenchymal thinning, and recommended acquiring multiple images. Song C et al. [36] examined post-pyeloplasty function in UPJS patients with supranormal function and found that a significant decrease in function was only associated with renal parenchymal thickness. There was no significant relationship between AP diameter, kidney size, and kidney size ratio. Parenchymal thinning has been reported to be associated with supranormal function and predict a >5% drop in postoperative SRF. However, we observed no relationship between parenchymal thickness and SRF assessed by Tc-99m MAG-3 and Tc-99m DMSA in our study. Studies have examined the relationship between supranormal function and kidney size, AP diameter, parenchymal thinning, and USG hydronephrosis grade, but their results have varied. These different results suggest that none of the parameters is an accurate indicator of supranormal function.

Parenchyma thickness is used in the classification of hydronephrosis and in surgical treatment decisions in patients with UPJS [5,18]. The renal parenchyma thins in patients with severe UPJS [21]. In our study, 35 patients (67.3%) exhibited thinning of the renal parenchyma (parenchyma thickness <7 mm). Additionally, when USG and Tc-99m MAG-3 results were compared in terms of parenchymal thinning, there was a statistically significant association (p=0.002), with 26 of 35 patients with parenchymal thinning on USG (83.9%) also assessed as having parenchymal thinning by Tc-99m MAG-3. The correlation between parenchymal thinning on USG and Tc-99m MAG-3 suggests that USG may be sufficient in monitoring whether parenchymal thinning of patients occurs.

Limitations and strengths

One of the limitations of our study was that it was a retrospective study and the number of cases was limited. However, the scintigraphy of the patients was re-evaluated by the same nuclear medicine specialist. Another limitation is that the number of patients with supranormal function is very small.

## Conclusions

The present study compared Tc-99m MAG-3 and Tc-99m DMSA scintigraphy in determining SRF, which guides the decision to pursue surgical or conservative treatment in children with UPJS. The results demonstrated 76% agreement in the SRF values obtained with the two methods. Although some studies have reported that Tc-99m MAG-3 causes supranormal function measurements in patients with UPJS, our results showed that Tc-99m DMSA resulted in a higher rate of supranormal values for affected kidneys, while Tc-99m MAG-3 scintigraphy detected more patients with low function. We believe that Tc-99m DMSA should not be performed in addition to Tc-99m MAG-3 scintigraphy in the follow-up of every patient with UPJS, but it can be utilized in select cases, such as patients with surgical indications and those suspected of needing surgery.
